# An Innovative One Health Approach: BIOQUALIM, a Transdisciplinary Research Action Protocol—From Cultivated Biodiversity to Human Health Prevention

**DOI:** 10.3390/nu16203495

**Published:** 2024-10-15

**Authors:** Audrey Murat-Ringot, Romain Lan, Laurie Fraticelli, Yohan Fayet, Denis Bourgeois, Rita Nugem, Maëva Piton, Emmie Goetz, Marie Préau, Fabien Dutertre, Nathalie Bernoud-Hubac, Lama Basbous, Anne Lastmann, Marie-Thérèse Charreyre, Florence Carrouel

**Affiliations:** 1 Laboratory “Health, Systemic, Process” (P2S), UR4129, University Claude Bernard Lyon 1, University of Lyon, 69008 Lyon, France; audrey.ringot@chu-lyon.fr (A.M.-R.); romain.lan@univ-lyon1.fr (R.L.); laurie.fraticelli@univ-lyon1.fr (L.F.); denis.bourgeois@univ-lyon1.fr (D.B.); rita-de-cassia.nugem@univ-lyon1.fr (R.N.); 2 Hospices Civils de Lyon, 69002 Lyon, France; lama.basbous@ripai.fr (L.B.); anne.silie@gmail.com (A.L.); 3 Laboratory Anthropologie Bio-Culturelle, Droit, Ethique et Santé (ADES), Aix Marseille University, Centre National de la Recherche Scientifique (CNRS), Etablissement Français du Sang (EFS), 13005 Marseille, France; 4 Department of Geography, Université Clermont Auvergne, AgroParisTech, INRAE, VetAgroSup, Territoires, 63000 Clermont-Ferrand, France; 5 Unité U1296 “Radiations: Défense, Santé, Environnement”, Université Lumière Lyon 2, 69007 Lyon, France; m.piton@univ-lyon2.fr (M.P.); emmie.goetz@univ-lyon2.fr (E.G.); marie.preau@univ-lyon2.fr (M.P.); 6 Universite Claude Bernard Lyon 1, INSA Lyon, Université Jean Monnet, Centre National de la Recherche Scientifique (CNRS), UMR 5223, Ingénierie des Matériaux Polymères, 42023 Saint Etienne Cédex, France; fabien.dutertre@univ-st-etienne.fr; 7 Laboratoire de Mécanique des Contacts et des Structures (LaMCoS), INSA Lyon, Centre National de la Recherche Scientifique (CNRS), UMR 5259, 69621 Villeurbanne, France; nathalie.bernoud-hubac@insa-lyon.fr; 8 Universite Claude Bernard Lyon 1, INSA Lyon, Université Jean Monnet, Centre National de la Recherche Scientifique (CNRS), UMR 5223, Ingénierie des Matériaux Polymères, 69622 Villeurbanne Cédex, France; marie-therese.charreyre@univ-lyon1.fr

**Keywords:** plant-based diet, planetary healthy diet, cancer, prevention, periodontal disease, einkorn, emmer, spelt, one health

## Abstract

Background/Objectives: The “One Health” approach underscores the connection between human, animal, and environmental health, promoting solutions to global challenges like climate change and biodiversity loss. The Planetary Health Diet (PHD) promotes a plant-based diet with organically grown plants to reduce the environmental impact of meat production and decrease the risk of non-communicable diseases (NCDs). The BIOQUALIM project will evaluate the PHD’s effectiveness in preventing NCDs like periodontal diseases and cancers through four inter-related studies. Methods: The clinical study will involve volunteers reducing their meat consumption and incorporating einkorn into their diet, allow for analysis of their interdental microbiota, oral health, general health, and quality of life. The chemical analysis will study nutrients and anti-cancer compounds in einkorn and common wheat varieties. The behavioral study will explore PHD knowledge, attitudes, and behaviors related to PHD. The psycho-social study will evaluate the impact of peer-support workshops on plant-based dietary cooking among post-therapy cancer patients. Results: The results are expected to demonstrate that einkorn varieties possess nutritional properties that, when incorporated into the PHD enriched with einkorn, can enhance health markers. This study will identify barriers to and facilitators of PHD adoption and highlight how peer-support workshops can improve dietary adherence. Conclusions: BIOQUALIM’s transdisciplinary approach will demonstrate the PHD’s role in preventing NCDs.

## 1. Introduction

The “One Health” concept highlights the interdependence of human, animal, plant, and environmental health [1]. It is a holistic approach aimed at addressing global challenges such as antimicrobial resistance, food insecurity, and climate change [2]. The COVID-19 pandemic underscored the need for integrated, cross-disciplinary strategies to prevent, detect, and manage diseases that emerge from disrupted ecosystems [3]. Human health is particularly vulnerable in this interconnected system, with human activities like industrialization and natural resource exploitation playing a significant role in upsetting the balance [4]. These disruptions lead to the emergence of zoonotic diseases, antibiotic resistance, and other health issues. Transdisciplinary collaboration is crucial for addressing these challenges of the 21st century, underscoring the relevance of the “One Health” concept [5].

The Planetary Health Diet (PHD), proposed by the EAT-Lancet Commission, is an integral part of the One Health framework, promoting a diet that supports both human health and environmental sustainability [6,7]. The PHD emphasizes plant-based foods while reducing the intake of animal products, addressing the environmental and health risks associated with meat production [8]. Meat production has lasting effects on the environment, including deforestation, water scarcity, greenhouse gas emissions, and biodiversity loss [9,10]. It also contributes to human health risks through the potential for zoonotic diseases, as 60% of human pathogens and 75% of emerging infectious diseases originate from animals [11]. Biodiversity loss further exacerbates these challenges by weakening ecosystem resilience and limiting opportunities for medical discoveries from nature [12].

Studies indicate that dietary changes are crucial for improving both human health and environmental sustainability [13,14,15,16]. High meat consumption and industrialized foods, prevalent in high-income countries, are linked to higher cancer rates and other non-communicable diseases (NCDs), such as diabetes and cardiovascular conditions [17,18,19]. This is due to the nutritional imbalances caused by diets high in refined grains, salt, saturated fats, and sugars, and low in fruits and vegetables. Meat consumption may promote carcinogenesis, including chronic inflammation, the production of toxic compounds during high-temperature cooking, hormonal imbalance, and consequences of intestinal dysbiosis [20,21,22,23].

Conversely, plant-based diets, including the PHD, Mediterranean diet, and Dietary Approaches to Stop Hypertension diet, offer significant health benefits [6,24,25]. These diets, rich in fiber and low in saturated fat, reduce the risk of obesity [26,27], type 2 diabetes [24,28,29], cardiovascular diseases, and some cancers [29,30,31]. They are also more environmentally sustainable, addressing global health and ecological challenges simultaneously by promoting better dietary practices and reducing environmental impact [32].

Reducing animal proteins and increasing plant proteins in diets, especially in industrialized countries, addresses both public health and environmental concerns [33], with plant proteins being more cost-effective [34]. However, declining biodiversity restricts the variety of available plant protein sources, affecting the ability to meet recommended amino acid intake [35]. Studies have revealed chronic micronutrient deficiencies due to the declining nutritional quality of fruits, vegetables, and grains, driven by reduced crop diversity and industrial agricultural practices [36,37,38]. However, “ancient wheat” species like einkorn, emmer, and spelt, though less common, offer superior nutritional benefits [39,40] and higher levels of bioactive compounds with anti-cancer properties, such as phytosterols [41].

An important area of focus is the link between diet, microbiota, and NCDs. A plant-based diet rich in proteins and fibers and low in sugars and saturated fats, such as the PHD, could help prevent dysbiosis of the intestinal and oral microbiota, a condition linked to NCDs such as periodontal diseases and cancers [19,42,43,44]. Pathogenic oral bacteria, including *Porphyromonas gingivalis* and *Treponema denticola*, are risk factors for periodontal diseases, which are in turn connected to systemic conditions [42]. Controlling microbiota dysbiosis through diet offers the potential to prevent a range of NCDs. While the PHD may help reduce oral dysbiosis, more research is needed to explore its specific role in preventing periodontal diseases [45] and other NCDs [19].

**Figure 1:**
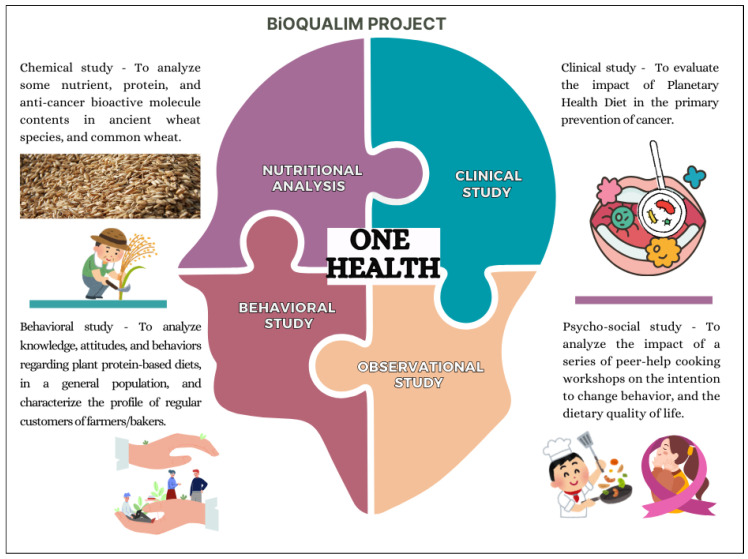
The studies of the BIOQUALIM project. Made by the authors from a Canva model.

The BIOQUALIM project applies the One Health approach by assessing the PHD’s role in preventing NCDs, particularly periodontal diseases and cancers. The project includes four complementary studies: clinical, chemical, behavioral, and psycho-social (Figure 1). The clinical study will evaluate how the PHD affects oral microbiota dysbiosis and participants’ quality of life. The chemical study will examine the nutritional properties of various wheat species, which will be key components of the PHD for participants in the clinical study and in culinary workshops as part of the psycho-social study. The behavioral study will identify the barriers and motivators for adopting the PHD, which is essential for encouraging broader dietary change. Western societies often face cultural, economic, and convenience-related challenges to reducing meat consumption. Understanding these factors will help design interventions that promote healthier eating habits. The psycho-social study will focus on peer-assisted culinary workshops for cancer patients, helping them adopt the PHD as part of tertiary prevention. These workshops will offer support for participants to learn how to prepare plant-based meals, facilitating long-term behavior change.

Together, the four studies of the BIOQUALIM project aim to provide a comprehensive understanding of how the PHD can contribute to preventing NCDs and improving health outcomes. This aligns with national health initiatives like the French National Environmental Health Plan and the French National Nutrition and Health Program [41,46,47,48,49] and internationals guidelines including those from the WHO [50] and FAO [51], both of which emphasize sustainable eating patterns for human and environmental health. The BIOQUALIM project demonstrates how sustainable dietary practices can address the global burden of NCDs while promoting environmental sustainability.

## 2. Materials and Methods

### 2.1. Main Features of BIOQUALIM Project

**Figure 2:**
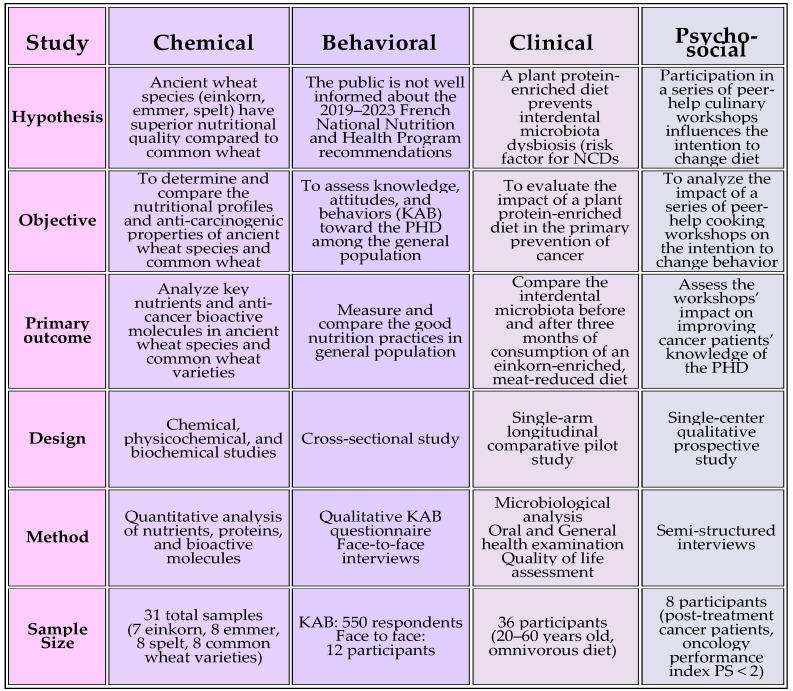
Summary of BIOQUALIM project.

The BIOQUALIM project (Figure 2) is a national project supported by a grant from the French National Research Agency as part of the “Investissements d’Avenir ExcellencES” program from France 2030 (SHAPE-Med@Lyon; ANR-22-EXES-0012). This project started in December 2023 and will finish in December 2025.

The BIOQUALIM project will be managed by six French research teams developing a transdisciplinary approach: (i) the laboratory Polymer Materials Engineering (IMP) belonging to the University Claude Bernard Lyon 1, the National Institute of Applied Sciences (INSA), and the National Center for Scientific Research (CNRS, Paris, France), (ii) the Laboratory Systemic Health Pathway (P2S) UR4129 belonging to the University Claude Bernard Lyon 1, (iii) the Cancer Coordination Center (3C-HCL) belonging to the Civil Hospitals of Lyon, (iv) the laboratory Contact and Structural Mechanics (LaMCoS, Lyon, France) belonging to INSA and CNRS, (v) the UMR Territoires and the Department of Geography-Planning belonging to the University Clermont-Auvergne, and (vi) the Center for Social Psychology (PoPS) INSERM U1296 belonging to INSERM and the University Lyon 2. In addition, other partners will contribute: the School of Engineering in Agronomy, Agri-food, and Environment (ISARA, Lyon, France), the National Research Institute for Agriculture, Food, and the Environment (INRAE, Paris, France), and the Center for Applied Botany Resources (CRBA).

This transdisciplinary approach enables the combination of diverse expertise to address the issues from different angles, ensuring a comprehensive and integrated understanding of the challenges related to food, health, and the environment.

The methodological approach is based on action research, with the involvement of a diverse range of stakeholders, including civil society actors (such as a seed conservatory, collectives of partner farmers, collective hospital catering services, and partner patients from Lyon Civil Hospitals) and research teams from complementary disciplines (including chemistry, biochemistry, microbiology, public health, geography, social psychology). This collaborative framework aims to integrate practical and scientific expertise to address the project goals effectively.

### 2.2. Evaluation of the Impact of the Planetary Health Diet on Interdental Microbiota Dysbiosis, a Risk Factor of NCDs Such as Cancer, and on Quality of Life in the General Population (Clinical Study)

#### 2.2.1. Study Design

**Figure 3:**
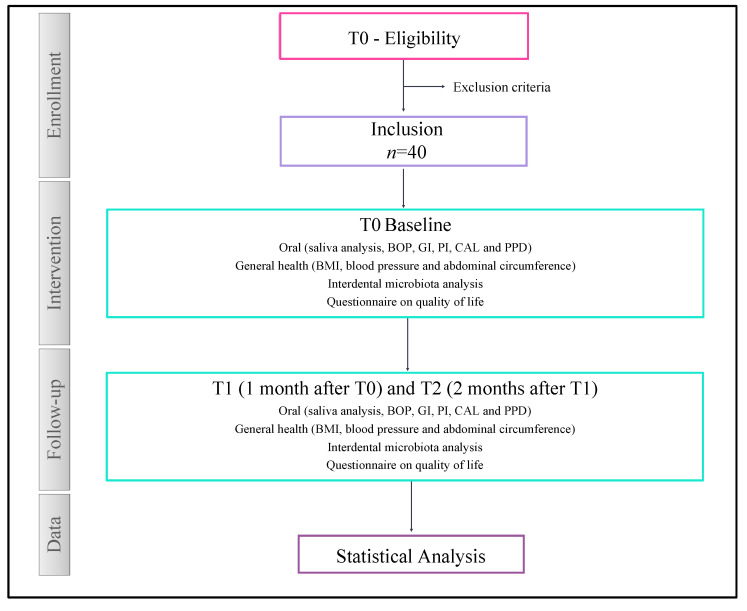
A flow chart diagram of the “clinical study”. BOP: Bleeding On Probing, GI: Gingivitis Index, PI: Plaque Index, CAL: Clinical Attachment Level, PPD: Probing Pocket Depth, and BMI: Body Mass Index.

The “clinical study” will be a single-arm longitudinal comparative pilot study (Figure 3). The protocol will follow the Standard Protocol Items: recommendations for international trials (SPIRIT) [52].

#### 2.2.2. Study Setting

The “clinical study” will be conducted between January 2025 and December 2025 at the HCL (Lyon, France).

#### 2.2.3. Outcomes

The primary outcome will be to compare the number of total bacteria and periodontal pathogens of the interdental microbiota before (T0) and after 3 months (T2) of consumption of a diet enriched in cereal (einkorn) and reduced in meat.

The secondary outcomes will be as follows:

- To compare the number of total bacteria and periodontal pathogens of the interdental microbiota before (T0) and after 1 month (T1) of consumption of a diet enriched in einkorn and reduced in meat.
- To compare periodontal and oral clinical parameters before (T0), during (T1), and after (T2) the introduction of einkorn into the diet.
- To compare general health indicators before (T0), during (T1), and after (T2) the introduction of einkorn into the diet through a consultation and a general medical examination.
- To compare quality of life before (T0), during (T1), and after (T2) the introduction of einkorn into the diet through answers to a questionnaire.

#### 2.2.4. Study Population and Inclusion Criteria

This clinical study will include people who (i) are between 20 and 60 years of age, (ii) have an omnivorous diet, (iii) have a body mass index greater than 18 kg/m^2^, and (iv) have given written consent. The exclusion criteria are as follows: (i) a smoking addiction, (ii) a known food allergy/intolerance, (iii) an inability to consume dairy or solid products, (iv) a high risk of infective endocarditis, (v) chronic pathologies, (vi) antibiotic treatment during the month preceding the start of the study, (vii) pregnancy in progress, parturient, or breast feeding, (viii) deprivation of liberty by judicial or administrative decision, (ix) psychiatric care, (x) legal protection (guardianship, trusteeship), (xi) having less than 20 natural teeth, with active cavities, (xii) performing mouthwashes or interdental hygiene regularly, and (xiii) using orthodontic braces.

#### 2.2.5. Sample Methodology

Participants will be recruited using (i) a database of volunteers who have already participated in other studies for the P2S laboratory and who have indicated their wish to participate in other similar studies, (ii) a call for participation by an email sent to all HCL staff, and (iii) a call for participation broadcast on the social networks of the P2S laboratory.

#### 2.2.6. Sample Size

The research hypothesis is part of a superiority test (fewer pathogenic bacteria after changing diet versus before). Referring to the results obtained by Bourgeois et al. [53], with a statistical power of 90% and an expected reduction difference at 3 months of 0.5, 36 participants must be included for significance with a *p*-value < 0.05. Considering an estimated dropout rate of 10%, we plan to include 40 participants. If there are larger dropouts or exclusions, recruitment of additional volunteers will be implemented to ensure the inclusion of 36 participants.

#### 2.2.7. A Description of the Procedure and Data Collection

The participants will have to change their diet for 3 months. They will reduce their meat consumption by half and will consume einkorn (at least 100 g/day of cooked whole grains, 6 days/week). To achieve this, cooking workshops will be offered during the 3 months of the study.

Three visits will be planned during the study, (T0 (inclusion), T1 (1 month after T0), and T2 (2 months after T1)), to collect the following data:

- Microbiological: Sampling of interdental microbiota [54,55,56,57] and real-time PCR to quantify the total number of bacteria and 9 periodontal bacteria. Among them, several are considered cancer risk factors: *Aggregatibacter actinomycetemcomitans*, *Porphyromonas gingivalis*, *Tannerella forsythia*, *Treponema denticola*, *Prevotella intermedia*, *Parvimonas micra*, *Fusobacterium nucleatum*, *Campilobacter rectus*, and *Eikenella corrodens*.
- Oral health: Quantitative and qualitative analysis of saliva (GC saliva check buffer, GC, Sucy-en-Brie, France) and measurement of periodontal parameters (Bleeding On Probing (BOP) [58], Gingivitis Index (GI) [59], Plaque Index (PI) [60], Clinical Attachment Level (CAL), and Probing Pocket Depth (PPD) [61].
- General health: Body Mass Index (BMI), blood pressure, and abdominal circumference.
- Quality of life: MOS SF-36 self-questionnaire validated in French [62].

#### 2.2.8. Data Analysis

Quantitative data encompassing the entire population will be described, including measures of central tendency and dispersion such as mean, standard deviation, and median. Qualitative variables will be summarized, detailing frequencies and percentages for each level, accounting for missing values without including them in frequency calculations. Absolute and relative frequencies will be calculated for the entire population and at multiple time points (T0, T1, T2). A linear regression analysis will assess the relationship between the interdental microbiome and oral and general health indicators. Paired *t*-tests will compare relative amounts of total and pathogenic bacteria between T0, T1, and T2. Statistical significance will be set at α = 0.05. The analysis of health indices, anthropometric measures, health parameters, and quality of life will employ analysis of variance (ANOVA) to compare trends over time. Linear mixed models could evaluate the impact of dietary intervention on the interdental microbiome, treating time as a random variable and covariates as fixed variables.

Intention-to-treat analyses will be performed on imputed data from all randomized patients using multiple imputation by chained equations, based on a chained Monte-Carlo–Markov algorithm under the assumption of missing data at random.

Descriptive statistics will be calculated with SPSS 12.0 (SPSS Inc., Chicago, IL, USA). Statistical tests (*p*-values) will be calculated with SUDAAN 7.0 (Research Triangle Institute, Research Triangle Park, NC, USA) for repeated measurements (measurements throughout the follow-up period).

### 2.3. Characterization of the Nutritional Properties of Cereals, Especially Ancient Wheat Species (Chemical Study)

#### 2.3.1. Study Design

The experiments will be conducted from November 2024 to December 2025 at (i) the Contact and Structural Mechanics Laboratory (LaMCoS), which features a state-of-the-art lipidomics platform, recognized with the IBiSA label (Infrastructures in Biology, Health, and Agronomy) and ISO9001-certified; and (ii) the Polymer Material Engineering laboratory (IMP).

#### 2.3.2. Outcomes

The primary outcome of this “chemical study” of cereal properties will be to analyze some nutrient and anti-cancer bioactive molecule contents and to study proteins in ancient wheat species, einkorn (*Triticum monococcum* ssp. monococcum), emmer (*Triticum turgidum* ssp. dicoccum), and spelt (*Triticum aestivum* ssp. spelta), in comparison to common wheat species (soft wheat, *Triticum aestivum* spp. aestivum). The secondary outcomes will be to (i) compare the composition between the three ancient wheat species and with common wheat and (ii) evaluate the differences in terms of varieties within each species.

#### 2.3.3. Samples

Twenty-three varieties of ancient wheat species (seven varieties of einkorn, eight varieties of emmer, eight varieties of spelt) and eight varieties of common wheat (soft wheat), which were grown organically in the Lyon region (France) at the Centre de Ressources de Botanique Appliquée (CRBA, Charly) and harvested during summer 2022, will be used. Several of these varieties have also been grown by 6 farmer-bakers involved in the study and located within an 80 km radius around Lyon. The majority of these cereals are local varieties, easily accessible to farmers–bakers in the Lyon region (the seeds came from farms or associations located in the center-east of France).

#### 2.3.4. Chemical Analysis

The quantitative analysis of nutrients and bioactive molecules will be carried out by different methods, in particular inductively coupled plasma mass spectrometry (ICP-MS) and gas chromatography coupled with tandem mass spectrometry (GC-MS/MS). The protein study will be performed combining various physicochemical techniques, such as size exclusion chromatography, light scattering, and rheology.

### 2.4. An Investigation of the Plant-Based Food Consumption Perceived Behaviors Captured in the General Population (Behavioral Study)

#### 2.4.1. Study Design

The “behavioral study” is a mixed cross-sectional study, employing both quantitative and qualitative methodologies. It will be conducted in two steps.

The quantitative phase will involve a survey to assess knowledge, attitudes, and behaviors (KAB) related to the PHD in the general population. The survey will be structured with a total of 35 items (1 item with an open-ended answer and 34 items with closed-ended answers).

This qualitative phase will focus on face-to-face interviews with regular customers of farmers–bakers. These interviews will allow for a deeper exploration of the motivations, behaviors, and profiles of these customers.

Both phases will contribute to a comprehensive understanding of the KAB concerning the PHD, with the quantitative data providing generalizable results and the qualitative interviews offering nuanced insights into the specific population of regular customers of farmers–bakers.

#### 2.4.2. Study Setting

The analysis of the KAB regarding the PHD, the first step of the “behavioral study”, will be conducted between March 2024 and December 2025.

The face-to-face interviews, the second step of the “behavioral study”, will be conducted between March 2024 and May 2024.

#### 2.4.3. Outcomes

The main objective will determine the percentage of people in the general population with attitudes and behaviors corresponding to the PHD.

The secondary outcomes will be as follows:

- To assess the general population’s knowledge of the PHD;
- To identify determinants of the respondents with attitudes and behaviors corresponding to the PHD;
- To identify obstacles and levers among regular consumers of farmers–bakers regarding their consumption of plant-based foods.

#### 2.4.4. Study Population and Inclusion Criteria

For the KAB questionnaire, the target population will be (i) people over 18-years-old, (ii) people able to read and understand the French language, (iii) users (patients and professionals) of the central kitchen of the Hospices Civils of Lyon (HCL), and (iv) customers of farmers–bakers.

For the face-to-face interviews, the target population will be the regular customers of farmers–bakers.

#### 2.4.5. Sample Methodology

For the KAB questionnaire, the population will be recruited on the basis of a convenience sampling methodology by distributing the questionnaire to users of the central kitchen of the HCL and to customers of farmers–bakers.

For the face-to-face interviews, the target population will be recruited thanks to the farmers–bakers already identified in the chemical study as suppliers of ancient wheat species (einkorn, emmer, spelt) and common wheat.

#### 2.4.6. Sample Size

For the KAB questionnaire, considering a population of more than 10,000 users of the HCL central kitchen, with a confidence level of 95% and a margin of error of 5%, an estimated return of 383 questionnaires is deemed representative of the population.

For the face-to-face interviews, it is recognized that in the case of a homogeneous sample, (i) the first five to six participants produce the majority of new information in the dataset, while little information is obtained from subsequent participants; (ii) data from the first ten participants identify 80–92% of the information [63], and when the sample size is close to 20 interviews, little new information will be collected [64]. Thus, about 12 participants should allow for data saturation [65].

#### 2.4.7. A Description of the Procedure, Data Collection, and Interviews

For the KAB questionnaire, a self-administered questionnaire will be developed by the research team in a transdisciplinary manner based on the Food Choice Questionnaire [66]. The KAB questionnaire will be tested by 10 persons who will not be included in the final study. The questionnaire will take an estimated 12 min to complete. It will be available in paper or digital version with a QR code accessible on a tablet, PC, or smartphone (Supplementary File S1). All questions are mandatory.

For the face-to-face interviews, participant experiences will be collected by a qualitative study specialist using a semi-structured individual interview guide. This guide will be developed specifically because no pre-used or validated models were identified in the literature. Therefore, it was conceived by the research team composed of specialists in public health and psycho-social sciences and students in agronomy. Each member established a list of relevant questions, and then all proposals were pooled and discussed until consensus was reached on the questions to include in the interview guide. The guide will be tested during two interviews with individuals from the studied population who are not participating in the final study. This test will evaluate the time needed for the interview, check the reliability and understanding of each question, and adapt the guide if necessary. The interview guide will be composed of four sections: (i) knowledge of nutrition, (ii) plant-based protein intake, (iii) eating habits, and (iv) changes in eating habits.

#### 2.4.8. Data Analysis

A univariate analysis will describe all of the characteristics of the respondents of the KAB study including socio-demography, income level, and place of residence. Some covariates of interest will be identified for stratification (e.g., age group). The sub-groups will be compared to identify major differences or similarities. A multivariate analysis will evaluate the determinants of the outcomes (K, A, and B), especially the socio-spatial disparities in terms of the PHD. Socio-demographic data (age, gender, socio-professional characteristics) and data relating to the place of residence will also be integrated (social deprivation, population density, type of agriculture in the municipality, and type of municipality thanks to the GeoClasH classification) [67]. The statistical analysis will be performed using the software SPSS 12.0 (SPSS Inc., Chicago, IL, USA).

The analysis of the face-to-face interviews will follow the methodological framework proposed by Braun and Clarke [68], which consists of a thematic analysis. First, the recordings of the conversations will be transcribed verbatim and checked to track the exact course of the interview, indicating all components of the communication to understand the interactions (including laughter, hesitations). Second, two researchers will carry out open coding with an in-depth reading of each verbatim transcription. They will use a mixed content analytic method: inductive and deductive. Third, the two researchers will discuss the initial codes until they reach an agreement and draw up the first codebook. In case of divergent opinions, a third researcher who is a member of the research team will be consulted. Fourth, researchers will use the structure of this codebook to analyze the remaining responses, remaining open to the inclusion of new codes or the refinement of existing ones. Fifth, the final structure with themes and subthemes will be finalized, and occurrences will be measured to weigh the results. Reliability during thematic data analysis will be ensured by systematically storing raw data, reporting detailed notes on the development and organization of concepts and themes, establishing consensus on the themes, providing precise descriptions of the context, and describing the coding and analysis process [69,70]. EXCEL^®^ 2019 MSO software will be used for data analysis.

### 2.5. Evaluation of the Impact of Health Promotion Programs Focusing on the PHD, Using Plant-Based Cooking Workshops to Provide Peer Support, on Cancer Patients (Psycho-Social Study)

#### 2.5.1. Study Design

The “psycho-social study” will be a cohort observational study that aims to determine if peer-support (led by a former patient) plant-based cooking workshops could be facilitators in promoting the PHD and reducing barriers to preparing and consuming it, specifically for cancer survivors.

#### 2.5.2. Study Setting

The “psycho-social study” will be conducted between February 2024 and December 2025.

#### 2.5.3. Outcomes

The primary outcome will be to analyze the impact of peer-support plant-based cooking workshops on improving post-treatment cancer patients’ knowledge of the PHD and their intention to introduce protein-rich cereals (einkorn, emmer, and spelt) into their diet.

The secondary outcomes will be as follows:

- To analyze the satisfaction of post-treatment cancer patients regarding the introduction of protein-rich cereals into their diet;
- To raise patients’ awareness of micro-environmental health (reading labels, the benefits of plant-based food rich in protein and grown organically) and macro-environmental health (agricultural systems and their link with health, One Health approach);
- To analyze the perception of social support and the knowledge and confidence gained through the cooking workshops.

#### 2.5.4. Study Population

This “psycho-social study” will include participants who (i) are over 18 years of age, (ii) have suffered from cancer in all specialties and are post-therapeutic (<2 and 5 years after the end of treatment for solid tumor and hematology, respectively), and (iii) have an oncological performance status (PS) assessment <2 [71]. The exclusion criteria will be as follows: (i) to present contraindications to the consumption of whole grains, pulses, fruits, and vegetables and (ii) to be unavailable on the workshop dates.

#### 2.5.5. Sample Size

To optimize the intervention experience and maintain sufficient interactivity between all members of the group that will be led by two patient partners, groups of 8 participants will be set up. Participants will be recruited through purposive sampling [72].

#### 2.5.6. A Description of the Procedure and Data Collection

Participants will participate in six 2-h themed cooking workshops spaced 2 weeks apart in a specially equipped therapeutic kitchen (4). During each workshop, two recipes will be performed using either einkorn, emmer, or spelt in different forms (whole grains, couscous grains, flakes, flour, and crushed grains). An educational summary sheet and recipe sheets will be provided to each participant at the end of each workshop.

For these cooking workshops, an evaluation process based on the Kirkpatrick model [73] will be used to evaluate the health benefits of these workshops, according to the 4 levels described below.

Level 1 will evaluate the immediate reaction of participants (pedagogical evaluation of the workshops) via one questionnaire given at the end of each workshop and face-to-face interviews (Supplementary File S2). This questionnaire will be composed of seven questions (six closed questions and one open question).

Level 2 will evaluate the acquisition of knowledge/skills (food literacy, in particular the ability to find information on food and cancers, to understand it critically; “empowerment”, in particular increased confidence regarding the consumption of plant proteins and one’s own cooking skills). Participants will answer one questionnaire before and at the end of the cooking workshops series composed of 13 closed questions (Supplementary File S3).

Level 3 will evaluate the modification of cooking and consumption behavioral habits, whereas level 4 will evaluate patient satisfaction with (i) their general state of health (Patient-Reported Experience Measures, PREMs) [74] and with (ii) the adequacy of these cooking workshops in relation to their needs at the end of their treatment (tertiary prevention) in order to ensure the reproducibility of the workshops. These two levels will be evaluated during semi-structured individual interviews. The interview guide was developed for this study by the research team on the consensus principles. This interview guide (Supplementary File S4) is composed of four sections: (i) the “theme of cancer food” that aims to explore food representations by cancer patients, (ii) the “benefits of cooking workshops” that aims to identify perceived social support, self-efficacy, and representations about the group dynamics, (iii) the “after the cooking workshops” that aims to analyze behavioral intention to introduce einkorn, emmer, or spelt cereals into eating habits, and (iv) the “final questions” that aims to collect satisfaction level, suggestions, and opinions about the workshops. This interview guide will be tested during one interview with a patient belonging to the study population but not participating in the study. This test will assess the time needed for the interview and verify the consistency and the good comprehension of each question, enabling adjustments if necessary. To consolidate knowledge input, participants will be invited to attend a seminar after the six workshops where a specialist will provide information about these protein-rich cereals.

#### 2.5.7. Data Analysis

The analysis of questionnaires will be descriptive and qualitative. The focus of descriptive analysis is to understand whether, behind one or more recurring phenomena, there are trends or patterns that can be mapped out (percentage, mean, etc.). The qualitative analysis will compare the questionnaires to identify patterns, similarities, and significant differences in the data, contributing to a deeper understanding, with valuable information.

The analysis of the face-to-face interviews will follow the same methodology as the analysis of the face-to-face interviews from the behavioral study. Excel^®^ software will be used to establish a coding tree and then determine the different themes to classify all verbatims.

## 3. Results and Discussion

The BIOQUALIM project is an action research project where researchers from various disciplines work together with citizens, especially patient partners, that have been involved from the conception to the different steps of the project. Firstly, the results of the “chemical study” will permit us to characterize the nutritional properties of cereals, especially ancient wheat species, and should highlight their high nutritive potential and their content of bioactive compounds with anti-cancer properties. Secondly, the results of the “behavioral study” will help to characterize the profile of PHD consumers and the lever and barriers to the adoption of the PHD. Thirdly, the results of the “clinical study” will permit us to determine if the PHD could reduce the dysbiosis of the oral microbiota, a risk factor for NCDs, and thus prevent them. Fourthly, the “psychological study” will determine the impact of health promotion programs focusing on the PHD through peer support on cancer patients. Thus, the results of the BIOQUALIM project could offer potential recommendations for policy and practice that support the implementation of the PHD for the primary prevention of periodontal disease and cancer, as well as tertiary cancer prevention. More generally, BIOQUALIM could re-emphasize the importance of a One Health approach for human health and a change in food production and consumption behaviors.

Concerning the nutritional and chemical properties of ancient wheat species, einkorn and spelt are noted for their high antioxidant capacity, which contributes to their potential health benefits [75]. Research has demonstrated that cookies enriched with einkorn exhibit superior physicochemical and nutritional properties compared to those made with regular wheat, suggesting that einkorn could enhance the health-promoting attributes of cereal-based products [76]. Additionally, studies have highlighted the potential of ancient wheat species, such as einkorn, to improve the nutritional content of modern wheat [77,78,79]. While these findings emphasize the nutritional advantages of ancient species, they do not specifically address the prevention of NCDs, which is the focus of this study. More recently, Kliemann et al. (2023) have investigated the role of ancient wheat species like einkorn in cancer prevention [78]. Although research directly linking einkorn to cancer prevention is still emerging, there is substantial evidence suggesting that whole grains, rich in fiber, vitamins, and antioxidants, may reduce the risk of certain cancers, particularly colorectal cancer. The European Prospective Investigation into Cancer and Nutrition (EPIC) study [80], the report from the International Agency for Research on Cancer (IARC) [81], and relevant recent studies have highlighted the protective role of dietary fiber from whole grains in reducing cancer risk, especially in colorectal and breast cancers [80,82,83]. Furthermore, evidence suggests that minimally processed grains, such as einkorn, could be more effective in cancer prevention compared to highly processed foods [77,84].

The BIOQUALIM project will have several perspectives: From the point of view of physical chemistry and biology, depending on the “chemical analysis” results, a study of the digestibility of plant proteins according to the type of cooking process used, including a physicochemical study of the evolution of the state of aggregation of these proteins before and after cooking, could contribute to developing suitable and healthier cooking procedures. From the point of view of educational sciences and public health, depending on the “chemical analysis” and “behavioral study” results, awareness programs could be developed to promote the most promising varieties of cereals and pulses which could enrich the cultivated biodiversity in the Lyon region (in organic farming). The implementation of targeted health promotion actions following the study on the extent of socio-spatial disparities in terms of eating habits and knowledge could contribute to reducing territorial health inequalities. In addition, it could be interesting to construct a system for the primary prevention of cancers in public health through education and health promotion of food rich in plant proteins (from the field to the plate). Hence, the results of the behavioral study and the psycho-social study could be used to develop education and training programs (continuing training catalog for health and school establishments). Moreover, an epistemological approach, focusing on the benefits of establishing a pro-literacy environment in the context of food literacy as defined by Slater [79], could be applied. This pro-literacy environment corresponds to an applied philosophy study that aims to more effectively translate scientific findings from several disciplinary fields through active participation and the integration of stakeholders’ concerns and expectations. From the point of view of microbiology, metabolomics, and public health, if the pilot clinical study leads to significant results, a mixed methodology could be used. For the quantitative part, a three-arm randomized controlled trial (control group/meat-depleted diet/meat-depleted diet supplemented with einkorn) will be set up, with analysis of the oral and fecal microbiota (by sequencing) and correlation with diet. For the qualitative part, semi-structured interviews or focus groups could be conducted and analyzed. From the point of view of economy and public health, health economics studies could be carried out to answer questions of social justice about the consumption of cereals and pulses from local organic farming. The results of the BIOQUALIM project will be disseminated through peer-reviewed journals, conferences, and public seminars to ensure broad access to the research outcomes. Throughout the project, we will engage with key stakeholders, including participants, healthcare professionals, and policymakers, to facilitate the translation of the research findings into practice and policy.

The BIOQUALIM project has several limitations. Firstly, the contribution of plant proteins to the diet is focused on einkorn in the clinical study and on ancient (einkorn, emmer, spelt) and soft wheat species in the chemical study, whereas many other sources of plant proteins exist. Secondly, the sample size is small and the single-arm design of the clinical study is a limitation, as the aim is to provide a proof of concept, since no data currently exist on the effect of diet modification on the oral interdental microbiota. Thirdly, the behavioral study will use convenience sampling which could induce some bias, even if it appears very low. Fourthly, the BIOQUALIM project is focused on ancient wheat species grown in the Lyon region (France), which has its own specificities (soils, climate, etc.). Fifthly, the questionnaires from the behavioral study will be realized online, which could result in misunderstandings of the questions due to the lack of direct supervision. Sixthly, in the clinical study, despite precautions and participants’ involvement in culinary workshops, there will be a possibility that some participants may not have adhered to the prescribed spelt consumption, potentially affecting dietary compliance. Finally, factors beyond diet, such as oral hygiene and physical activity, may also influence the oral microbiota, adding complexity to the clinical study’s outcomes.

## 4. Conclusions

The BIOQUALIM transdisciplinary project, based on a One Health approach, should provide valuable and innovative data on the impact of the PHD on the primary prevention of periodontal diseases and cancer, as well as on the tertiary prevention of cancer. According to the results, randomized controlled trials should be implemented to deepen the investigation of the impact of the PHD. In addition, socio-economic orientations should be strengthened to explore the conditions for the average cost of the healthy and environmentally sustainable food basket, for a family, composed of local, organic vegetables, pulses, and cereals, for example.

## Data Availability

The original contributions presented in the study are included in the article, further inquiries can be directed to the corresponding author.

## Supplementary Materials

The following supporting information can be downloaded at: https://www.mdpi.com/article/10.3390/nu16203495/s1, File S1. “Behavioral study” knowledge, attitudes, and behaviors questionnaire. File S2. “Psycho-social study” questionnaire to evaluate immediate reaction (level 1 of Kirkpatrick model). File S3. “Psycho-social study” questionnaire to evaluate knowledge and skills (level 2 of Kirkpatrick model). File S4. “Psycho-social study” face-to-face interview guide (level 3 and 4 of Kirkpatrick model).
