# Supplementary material for: An Innovative One Health Approach: BIOQUALIM, a Transdisciplinary Research Action Protocol—From Cultivated Biodiversity to Human Health Prevention
Source: Nutrients. 2024 Oct 15;16(20):3495. doi: 10.3390/nu16203495 (PMC11509970; doi:10.3390/nu16203495)
Supplement: Supplementary file 1 [file nutrients-16-03495-s001.zip › nutrients-3239220-supplementary.pdf]

Supplementary Material

An innovative one-health approach: BIOQUALIM, a transdisciplinary research-action protocol, from cultivated biodiversity to human health prevention

Audrey Murat-Ringot[1,2], Romain Lan[1,3], Laurie Fraticelli[1], Yohan Fayet[4], Denis Bourgeois[1], Rita Nugem[1], Maëva Piton[5], Emmie Goetz[5], Marie Préau[5], Fabien Dutertre[6], Nathalie Bernoud-Hubac[7], Lama Basbous[2], Anne Lastmann[2], Marie-Thérèse Charreyre[8], Florence Carrouel[1*]

Supplementary File S1 “Behavioral study” Knowledge, Attitudes and Behaviors questionnaire

*** All questions are mandatory ***

1. **You are** : ☐ MAN ☐ WOMAN ☐ OTHER

2. **Your age? _ _ years**

3. **Your department number _ _**

4. **The name of your town _**

5. **How many people are in your household, including yourself? _ _**

6. **What is your current occupation, or if retired/unemployed, what was your last occupation?**

   ☐ Farmer, operator

   ☐ Craftsman, shopkeeper, company director

   ☐ Executive and higher intellectual profession (liberal profession - doctor, lawyer, pharmacist, notary, etc. -, engineer, teacher, researcher, journalist, writer, etc.).

   ☐ Intermediate profession (teacher, nurse, educator, technician, inspector, supervisor, etc.)

   ☐ Employee (salesman, cashier, bank employee, civil servant, police officer, etc.)

   ☐ Worker

   ☐ Student

   ☐ Other (please specify if necessary)

7. **What is your main type of diet?**

   ☐ Omnivore

   ☐ Flexitarian (meat limitation)

   ☐ Pescovegetarian (no meat)

   ☐ Vegetarian (no meat or fish)

   ☐ Vegan (no animal products or derivatives)

   ☐ Other

8. **Do you, or anyone sharing your household, have any food allergies and/or intolerances, or a specific diet linked to a pathology (diabetes type for example)?**

	Allergy(ies)	Intolerance(s)	Special diet
You	☐**YES** ☐ **NO**	☐**YES** ☐ **NO**	☐**YES** ☐ **NO**
A member of your household	☐**YES** ☐ **NO**	☐**YES** ☐ **NO**	☐**YES** ☐ **NO**

9. **How much of your household budget do you spend on food?**
   - ☐ Less than 10 euros per week (less than 40 euros per month)
   - ☐ Between 11 and 20 euros per week (41 to 80 euros per month)
   - ☐ Between 21 and 50 euros per week (81 to 200 euros per month)
   - ☐ Between 51 and 100 euros per week (201 to 400 euros per month)
   - ☐ Between 101 and 150 euros per week (401 to 600 euros per month)
   - ☐ Between 151 and 200 euros per week (601 to 800 euros per month)
   - ☐ More than 201 euros per week (>800 euros per month)
   - ☐ I don't know

10. **Over 14 meals a week (lunch and dinner), how many meals do you or a member of your household cook on average? _ _**

11. **Of these cooked meals, how much time do you spend preparing them on average, per meal? _ _** in minutes

12. **How do you organize yourself to prepare meals in general (only one answer)?**
    - ☐ One person does the cooking for the home/accommodation
    - ☐ Everyone helps to make the meal
    - ☐ Everyone makes their own meal
    - ☐ Most of the time, meals are delivered to the home

2. Consumption habits

13. **Where do you do most of your <u>FOOD</u> shopping? 3 possible choices max**

☐	Supermarkets and hypermarkets
☐	Craftsmen and small retailers (bakeries, butchers, etc.)
☐	AMAP (Association pour le maintien d'une agriculture paysanne)
☐	Farms and local producers (organic or not)
☐	Specialty stores (organic or not)
☐	Markets (organic or not)
☐	Drive and internet (ORGANIC or not)

☐	Other :

14. For selected choices, please indicate average one-way travel time (in minutes)

Average time in minutes	
	Supermarkets and hypermarkets
	Craftsmen and small retailers (bakeries, butchers, etc.)
	AMAP (Association pour le maintien d'une agriculture paysanne)
	Farms and local producers (organic or not)
	Specialty stores (organic or not)
	Markets (organic or not)
	Drive and internet (ORGANIC or not)
	Other :

15. **What are your main criteria (max. 3 possible choices in order of preference) when choosing a store to buy from?**

☐	Prices
☐	Accessibility
☐	Habit
☐	Labeled products available (organic, Label Rouge, fair trade, etc.)
☐	Depending on advertising and promotions
☐	Store appreciation and reputation
☐	Variety of products sold
☐	Advice provided by staff
☐	Quality of products sold
☐	Fidelity cards and vouchers
☐	Other :

16. Do you know NUTRISCORE? ☐ yes ☐ no

16.1. If so, does this influence your purchasing decisions?

☐ Never ☐ Rarely ☐ Sometimes ☐ Often ☐ Always

17. **Do you use other resources to choose your products (phone applications, books, etc.)?** ☐ yes ☐ no

18. **What are your main criteria for choosing food products (max. 3 possible choices, in order of preference)?**

☐	Perceived effect on your health
☐	Origin
☐	Taste quality
☐	Price
☐	Habit
☐	Product composition (additives, sugar, salt, etc.)
☐	Environmental impact
☐	Product brand
☐	Labeled product (organic, red label, etc.)
☐	Producer remuneration and local employment
☐	Promotions/advertising
☐	Ready-made meals
☐	Animal welfare
☐	Other :

19. Do you ever buy produce from farms around your home? ☐yes ☐ no

20. Do you buy organic products?

☐ Yes, most of the time
☐ Yes, from time to time
☐ No, it's too expensive
☐ No, I can't find it where I shop
☐ No, I don't care
☐ No, I avoid it
☐ I don't know
☐ I never use it

21. Do you have a vegetable garden in your backyard?

☐ Yes, it's enough for me
☐ Yes, but I go shopping to supplement it
☐ No, but relatives regularly supply me with produce from their garden
☐ No

3. Overall consumption

19. In general, what foods do you eat? Several choices possible

FOODS	NEVER	RARELY	OCCASIONALLY	FREQUENTLY	VERY FREQUENTLY	No opinion
Fresh fruit (apples, bananas, oranges, etc.)						
Dried fruit (Raisins, dried apricots, prunes, etc.)						
Processed fruit (compotes, fruit in syrup, etc.)						
Vegetables (zucchini, carrots, tomatoes, green beans, sweet corn, peas, etc.) fresh, frozen and/or canned						
Dried vegetables (Lentils, chickpeas, split peas, broad beans, dried beans, lupin, soy (tofu, tempeh, textured soy protein, soy steak))						
Refined cereals (white bread and rusks, white pasta, white rice, white semolina, etc.)						
Semi-complete or complete wheat- and rice-based cereals (wholemeal bread and rusks, semi-complete pasta, semi-complete or complete pasta, semi-complete or complete rice, etc.)						
Non-wheat cereals and pseudo-cereals (spelt, oats, quinoa, millet, barley, buckwheat, rye, sorghum, etc.)						
Oilseeds (walnuts, hazelnuts, almonds, pistachios, seeds, etc.)						
Red meat (beef, pork, veal, mutton, lamb, goat, horse, wild boar, doe, etc.)						
White meat (chicken, turkey, rabbit...)						
Charcuterie and processed meats (cooked or raw ham, sausages and merguez, blood sausage, andouillette, sausage, bacon, corned beef, beef jerky, pâté, rillettes, kebabs, meatballs...)						
Oily fish (salmon, tuna, mackerel, sardines, herring)						
Other fish (cod, sea bass, sea bream, etc.)						
Seafood (mussels, shrimps, oysters, etc.)						
Dairy products (milk, yoghurt, cheese, cottage cheese, dairy products in ready-made meals)						
Eggs (eaten as is or in cakes, etc.)						
Animal fats (butter, cream, mayonnaise)						

Vegetable fats (vegetable mayonnaise, vegetable margarine)						
Oils rich in ALA (omega 3) (rapeseed oil, walnut oil, linseed oil, hemp oil, pumpkin seed oil, etc.)						
Oils low in ALA (sunflower oil, peanut oil, olive oil, sesame oil, grapeseed oil)						
Starch-based, processed salted/fatty products (French fries, potato chips, crackers)						
Sweet products (Cakes, pastries, cookies, chocolates, candies, sweetened breakfast cereals, sweetened milk desserts, ice creams, etc.)						
Industrial ready meals (pizzas, pasta, chili, lasagne and other reheatable dishes, packaged sandwiches, etc.)						
**DRINKS**	**NEVER**	**RARELY**	**OCCASIONALLY**	**FREQUENTLY**	**VERY FREQUENTLY**	**No opinion**
Alcohols (wine, beer, cider)						
Spirits (whisky, gin, vodka, digestif...)						
Sodas (soft drinks)						
Fruit juices (purchased)						
Home-pressed fruit juices						
Still water (tap or bottle)						
Sparkling water						
Filtered water						
Fermented drinks						
Herbal teas						
Tea						
Coffee						

20. In your opinion, does your food intake correspond to current nutritional recommendations?

☐yes ☐ no ☐ I don't know

21. Do you take pleasure in eating?

☐ Never or almost never
☐ Rarely
☐ Sometimes
☐ Often
☐Always or almost always
☐Don't know

4. Plant proteins, legumes and cereals

Launched by the French Ministry of Health and Prevention in January 2001, the overall aim of the **National Nutrition and Health Program** (“Programme national nutrition santé”, PNNS) is to improve the state of health of the entire population by acting on one of its major determinants: nutrition. At present, the PNNS aims to increase the population's consumption of legumes (at least two portions per week) while reducing that of red meat (less than 500g per week). The following questions focus on your knowledge of legumes.

21. Which of the following do you consider to be plant foods (multiple choices possible)?

☐ Nuts (peanuts, almonds, pistachios, walnuts, hazelnuts...)
☐ Green vegetables
☐ Lentils
☐ Eggs
☐ Cereals
☐ Fish
☐ Dried beans
☐ White meat
☐ Seafood
☐ Oils
☐ Dried peas (split peas, chickpeas...)
☐ Spelt
☐ Tofu
☐ Seeds (sunflower, pumpkin seeds, sesame...)
☐ Rice
☐ Quinoa
☐ Potatoes
☐ Buckwheat

22. Which of these foods are rich in vegetable protein (several choices possible)?

☐ Nuts (peanuts, almonds, pistachios, walnuts, hazelnuts...)
☐ Green vegetables
☐ Lentils
☐ Eggs
☐ Cereals
☐ Fish
☐ Dried beans
☐ White meat
☐ Seafood
☐ Oils
☐ Dried peas (split peas, chickpeas...)
☐ Spelt
☐ Tofu
☐ Seeds (sunflower, pumpkin seeds, sesame...)
☐ Rice
☐ Quinoa
☐ Potatoes
☐ Buckwheat

23. Do you consider that you are sufficiently informed about foods rich in plant proteins?

☐ not at all
☐ moderately
☐ quite
☐ don't know

24. Would you like more information about plant protein-rich foods?

☐ yes ☐ no

25. Do you usually eat pulses and/or legumes?

☐ Never or almost never
☐ Rarely
☐ Sometimes
☐ Often
☐ Always or almost always
☐ Don't know

26. Do you usually eat whole grains of small spelt and/or spelt?

☐ Never or almost never
☐ Rarely
☐ Sometimes
☐ Often
☐ Always or almost always

☐ Don't know

27. If yes, how do you consume these foods (pulses and/or legumes, whole grains of small spelt and/or spelt)?

	**Cooking (with water)**	**Germination**	**Fermentation**	**Precooked (quick cooking)**	**Already cooked (canned)**	**Other (please specify)**
Dried vegetables and/or pulses						
Whole grain spelt						

28. If you eat spelt, in what form (several answers possible)?

	**Whole seeds**	**Flour**	**Flakes**	**Pasta**	**Precooked (semolina, boulghour)**	**Other (please specify)**
Spelt						

29. Would you be willing to change your eating habits by replacing part of your meat portions with plant proteins?

☐ yes ☐ no ☐ don't know

30. If yes, which foods do you have in mind? free text_ _
_ _

Supplementary File S2 “Psycho-social study” Questionnaire to evaluate immediate reaction (level 1 of Kirkpatrick model)

At the end of each cooking workshop

1. For each statement, tick the box that most closely matches your feelings on this scale:
    1. Not at all satisfied
    2. Somewhat dissatisfied
    3. Moderately satisfied
    4. Quite satisfied
    5. Completely satisfied

My satisfaction with...	*Salty recipe*					*Sweet recipe*				
	1	*2*	*3*	*4*	*5*	*1*	*2*	*3*	*4*	*5*
Preparing the dish together										
Ease of preparation										
Time taken to prepare the recipe										
The visual aspect of the dish										
How it tastes										

2. Do you plan to reproduce one or both of the recipes you made at home today?

☐ yes ☐ no ☐ don't know

If not, what are the reasons?

………
………
………

3. What improvements would you like to see next time? (organization, duration, times, location, content, information provided, choice of recipes, entertainment, etc.)

………
………
………

Free comment:

………

……………………………………………………………………………………………………
……………………………………………………………………………………………………

Supplementary File S3 “Psychosocial study” Questionnaire to evaluate knowledge and skills (level 2 of Kirkpatrick model)

Before the beginning of the first cooking workshop

1. Have you ever taken part in cooking workshops?

☐ yes ☐ no

If yes, which theme(s) do you remember?
....

2. Do you have any expectations for the next 6 workshops?

☐ yes ☐ no ☐ don't know

3. If so, what do you expect from the next 6 workshops?

....

....

4. How old are you? ....

5. What's your gender?

☐ female ☐ male ☐ other ☐ do not wish to specify

6. In the course of your illness and treatment, what diet-related side effects have you encountered?

…………………………………………………………………………………………………

7. What do you know about spelt cereal?

Instructions: For each statement, check the box that most closely matches your situation on this scale:

1. Very low
2. Low
3. Medium
4. High
5. Very high

My level of knowledge about ... is ...	*1*	*2*	*3*	*4*	*5*
Ways to a balanced diet					
Spelt cereals in general					
The different types of spelt cereal (spelt, emmer, einkorn, etc.)					
The different forms of spelt cereal (flour, pasta, flakes, etc.)					

How to cook these types of cereals (hot, cold, sweet, savory, combined with other foods, etc.).					
How to promote good digestion when eating plant proteins such as spelt.					

8. And what about its cooking?

Instructions: For each statement, check the box that most closely matches your situation on this scale:

1. Strongly disagree
2. Somewhat disagree
3. Neutral
4. Somewhat agree
5. Completely agree

I feel capable of...	*1*	*2*	*3*	*4*	*5*
Cook a complete meal with spelt.					
Work on the presentation of a dish to make it appealing.					
Master the combinations of spelt to make it gourmet.					
Use different cooking techniques (cutting, soaking, cooking, deglazing, thickening a sauce, etc.).					
Master the different ways of cooking spelt.					
Design a recipe using spelt and create a shopping list accordingly.					
Find spelt and spelt-based foods in the shops.					

<u>At the end of the sixth cooking workshop</u>

1. Did participation in the workshops meet your expectations?

☐ yes ☐ no ☐ don't know

2. How old are you? ....

3. What do you know about spelt cereal?

Instructions: For each statement, check the box that most closely matches your situation on this scale:

6. Very low
7. Low
8. Medium
9. High
10. Very high

My level of knowledge about ... is ...	*1*	*2*	*3*	*4*	*5*
Ways to a balanced diet					
Spelt cereals in general					
The different types of spelt cereal (common spelt, medium spelt, etc.)					
The different forms of spelt cereal (flour, pasta, flakes, etc.)					

How to cook these types of spelt (hot, cold, sweet, savory, combined with other foods, etc.).					
How to promote good digestion when eating plant proteins such as spelt.					

4. And what about its cooking?

Instructions: For each statement, check the box that most closely matches your situation on this scale:

6. Strongly disagree
7. Somewhat disagree
8. Neutral
9. Somewhat agree
10. Completely agree

I feel capable of...	*1*	*2*	*3*	*4*	*5*
Cook a complete meal with spelt.					
Work on the presentation of a dish to make it appealing.					
Master the combinations of spelt to make it gourmet.					
Use different cooking techniques (cutting, soaking, cooking, deglazing, thickening a sauce, etc.).					
Master the different ways of cooking spelt.					
Design a recipe using spelt and create a shopping list accordingly.					
Find spelt and spelt-based foods in the shops.					

Supplementary File S4 "Psychosocial study" Face to face interview guide (level 3 and 4 of Kirkpatrick model)

Theoretical objectives: Verify the effectiveness of the cooking workshops by questioning the behavioral intention to adopt new eating habits regarding the spelt cereal. Question the acquisition of knowledge, skills, representation of group dynamics as well as perceived social support and potential development of a sense of self-efficacy during the workshops. Remain open to further information.

1 The theme of cancer food

"To begin with, we'll talk about the cancer diet in relation to your personal experience, and then return to the subject of cooking workshops"

Questions	*Reminders*
1. Since your illness, what has diet meant to you?	*How important is it in your life? How would you define food?*
2. Have you changed your diet since your illness?<br>If so, in what ways?	*Do you have a special diet? What do you cook most? What do you cook the least?*
3. Before the workshops, in what ways were legumes/cereals like spelt present in your diet?	*What did you know before the workshops? What did you cook? Did you know how to cook these foods? What did you think of these foods?*

2 The benefits of cooking workshops

"Now let's talk about what happened during the workshops and your personal experience"

Questions	*Reminders*
1. What attracted you to the workshops?	*What were your expectations?*
2. Would you say that the workshops met your expectations? In what way?	*Conversely, what did you feel was missing?*
3. Do you find it interesting to be in a group during the workshops? If so, which ones?	*How would you describe your interactions with the other participants? With the moderators? How would you describe your contribution? And that of others?*

4. In your opinion, did the workshops have an effect on what you know and can do?	*Do you see a difference between "before" and "after" the workshops? At what levels?*

3 After the cooking workshops

"Finally, let's talk about how you project yourself now that the workshops are over"

Questions	***Reminders***
1. Have you ever made any of the recipes seen during the workshops (or just spelt without a specific recipe) at home? Why or why not?	*What made you want to reproduce the recipes/cook spelt?*<br>*What prevented you from doing so?*<br>*What motivated or discouraged you to cook spelt?*
2. What did you think of these experiments? (If yes for Q n°1)	*Did you enjoy making the recipes again? Are you happy with the results? How was it different from the workshops?*
3. Have you shared what you got out of the workshops with anyone close to you? Tell me how it went.	*How did it make you feel? Are you satisfied with what happened? Are you thinking of doing it again?*
4. Do you intend to introduce spelt into your diet? Why or why not?	*What advantages do you see in introducing spelt into your diet? What are the drawbacks/obstacles?*
5. What would help you to introduce more spelt into your diet?	*How could it be more accessible for you? In terms of places, prices, recipe ideas...*

Final questions

1. Would you recommend these workshops to others?

2. Would you like to attend Ripaï workshops again? Why or why not?
